# Forecasting Daily Ambient PM_2.5_ Concentrations in Qingdao City Using Deep Learning and Hybrid Interpretable Models and Analysis of Driving Factors Using SHAP

**DOI:** 10.3390/toxics14010044

**Published:** 2025-12-30

**Authors:** Zhenfang He, Qingchun Guo, Zuhan Zhang, Genyue Feng, Shuaisen Qiao, Zhaosheng Wang

**Affiliations:** 1 School of Geography and Environment, Liaocheng University, Liaocheng 252000, China; guoqingchun@lcu.edu.cn (Q.G.); 2510140242@stu.lcu.edu.cn (Z.Z.); yue13847861122@163.com (G.F.); 13863531736@163.com (S.Q.); 2 Institute of Huanghe Studies, Liaocheng University, Liaocheng 252000, China; 3 State Key Laboratory of Loess Science, Institute of Earth Environment, Chinese Academy of Sciences, Xi’an 710061, China; 4 National Ecosystem Science Data Center, Key Laboratory of Ecosystem Network Observation and Modeling, Institute of Geographic Sciences and Natural Resources Research, Chinese Academy of Sciences, Beijing 100101, China; wangzs@igsnrr.ac.cn

**Keywords:** deep learning, ANN, RNN, CNN, transformer, BiLSTM, SHAP, PM_2.5_

## Abstract

With the acceleration of urbanization in China, air pollution is becoming increasingly serious, especially PM_2.5_ pollution, which poses a significant threat to public health. The study employed different deep learning models, including recurrent neural network (RNN), artificial neural network (ANN), convolutional Neural Network (CNN), bidirectional Long Short-Term Memory (BiLSTM), Transformer, and novel hybrid interpretable CNN–BiLSTM–Transformer architectures for forecasting daily PM_2.5_ concentrations on the integrated dataset. The dataset of meteorological factors and atmospheric pollutants in Qingdao City was used as input features for the model. Among the models tested, the hybrid CNN–BiLSTM–Transformer model achieved the highest prediction accuracy by extracting local features, capturing temporal dependencies in both directions, and enhancing global pattern and key information, with low root Mean Square Error (RMSE) (5.4236 μg/m^3^), low mean absolute error (MAE) (4.0220 μg/m^3^), low mean absolute percentage error (MAPE) (22.7791%) and high correlation coefficient (R) (0.9743) values. Shapley additive explanations (SHAP) analysis further revealed that PM_10_, CO, mean atmospheric temperature, O_3,_ and SO_2_ are the key influencing factors of PM_2.5_. This study provides a more comprehensive and multidimensional approach for predicting air pollution, and valuable insights for people’s health and policy makers.

## 1. Introduction

Air pollution affects ecosystems, climate change, food security, transportation, tourism, residents’ health, and sustainable economic development [1,2,3,4]. Air pollution has become a global environmental problem, with fine particulate matter (PM_2.5_) receiving widespread attention due to its serious harm to human health [5,6,7]. PM_2.5_ can rapidly enter the respiratory system, lungs, and bloodstream, significantly increasing respiratory and cardiovascular diseases, such as chronic bronchitis, emphysema, and lung cancer [8,9]. In 2019, the number of deaths due to exposure to ambient PM_2.5_ pollution in the world exceeded about 4 million, more than twice the total number of deaths reported in COVID-19 in 2020 (about 2 million) [10]. Sustainable Development Goal (SDG) 3.9 invites a significant reduction in global PM_2.5_-related deaths. Therefore, accurately predicting PM_2.5_ concentration is of great significance for developing effective air pollution control strategies and issuing health warnings.

Traditional air pollution prediction methods are mainly based on statistical models and numerical simulations, but these methods have limitations in dealing with complex nonlinear relationships [11]. Air pollution is influenced by temperature, humidity, wind speed, wind direction, air pressure, air pollutants (NO_2_, O_3_, and SO_2_), and spatiotemporal patterns [12,13,14]. Artificial intelligence (AI) has a powerful ability to handle nonlinear problems [15]. Deep learning (DL) technology has made significant progress in the field of time series forecasting [16,17,18,19]. An artificial neural network (ANN) is used to forecast daily PM_2.5_ concentrations in Ahvaz, Iran, with R (0.90) and RMSE (48.73) for the testing set [20]. The improved particle swarm optimized backpropagation neural network (IPSO-BP) ANN model is proposed to forecast daily PM_2.5_ concentrations in Nanchang City with the correlation coefficient R^2^ (0.9573), and the RMSE (5.2407) [21]. However, BP ANN has problems such as slow learning speed, easy to fall into local optima, and poor generalization ability.

The accuracy of the recurrent neural network (RNN) method is about 81% for PM_2.5_ forecasting, higher than the CMAQ (Community Multiscale Air Quality) forecast [22]. Two-State Gated Recurrent Unit (GRU) (Variants of RNN) is proposed for estimating the PM_2.5_ concentration in Florida with MAE (1.45), RMSE (3.48), and MAPE (16.80%) [23]. Similarly, as a variant, long short-term memory (LSTM) is developed for forecasting PM_2.5_ with (R^2^  =  0.973) in Delhi [24]. LSTM is also applied for PM_2.5_ prediction in India with a coefficient of determination (R^2^) (0.9) [25]. LSTM and GRU are applied to predict indoor PM_2.5_ levels, achieving RMSEs of 3.491 and 3.327, respectively [26]. A bi-directional LSTM (BiLSTM) network is proposed to predict long-term PM_2.5_ concentrations spatiotemporally with R^2^ (0.62), RMSE (12.887) [27]. The BiLSTM model outperforms other models (LSTM, GRU, TCN, ARIMA, and SARIMA) with R^2^ (0.947) and MAE (11.68) [28]. The accuracy of the convolutional neural network (CNN) for PM_2.5_ concentrations in Hefei exceeds that of the ANN by about 14.2% [29]. To better capture these relationships between PM_2.5_ concentrations and multiple variables, nine models are compared: RF (random forest), SVM (support vector machine), XGBoost (extreme gradient boosting), general regression neural network (GRNN), light gradient boosting machine (LGBM), DNN, adaptive boosting (Adaboost), DBN (deep belief networks), and transformer. The optimal model transformer is selected to estimate daily PM_2.5_ concentrations in Tianjin, with R^2^ (0.88), RMSE (15.30 μg/m^3^), MAE (9.55 μg/m), and MAPE (21.07 μg/m^3^) [30]. Transformer is constructed based on a self-attention mechanism, and has a powerful capability of modeling short-term and long-term dependencies of complex data [31]. Transformer has shown better performance than RNN and LSTM in capturing long-term dependencies in forecasting PM_2.5_ concentrations [32]. In summary, a single deep learning model can forecast PM_2.5_ concentrations.

Recently, hybrid models have achieved promising results in PM_2.5_ prediction tasks [33,34]. The bifold-attention LSTM (BA-LSTM) model is introduced to enhance PM_2.5_ forecasting accuracy in Beijing, achieving RMSE (0.013) and MAPE (3.891). BA-LSTM outperforms multilayer perceptron (MLP), LSTM, attention–LSTM, and attention BiLSTM [35]. A 1D-CNN–BiLSTM is constructed for hourly PM_2.5_ forecasting, with RMSE (3.88), MAE (2.52), and R^2^ (0.94) [36]. The PSO–CNN–BiLSTM model demonstrates strong short-term PM_2.5_ predictive capabilities with RMSE (16.73 μg/m^3^), R^2^ (0.84) in Jiaozuo City [37]. CNN–BiLSTM outperforms LSTM, CNN, and XGBoost. The multi-view stacked (MvS) CNN–BiLSTM outperforms RNN, GRU, and BiLSTM [38]. A hybrid CLSTM–GPR (gaussian process regression) model is used to forecast PM_2.5_ concentrations in Jiangmen and Huizhou, with R increasing by about 4.4% and RMSE decreasing by about 4.7% compared to LSTM–GPR, GPR, and CNN–GPR models [39]. STL–CNN–BILSTM–AM is introduced to forecast future PM_2.5_ concentrations in Delhi, India, with RMSE (3.51), MAE (2.52), and R^2^ (0.998) [40]. The R^2^ value of Transformer is about 30% higher than that of the CNN–LSTM–Attention [41]. The hybrid Transformer–BiGRU model is adopted to predict PM_2.5_ concentrations in Seoul subway stations with RMSE (2.03), MAE (0.56), and MAPE (1.6%) [42]. CNN–Transformer is established to predict high-resolution PM_2.5_ concentrations in Cangzhou with R^2^ (0.887) [43]. These studies have advanced the development of PM_2.5_ prediction by adopting hybrid models. The modeling combination of different deep learning architectures is the future research in this key field.

However, the “black box” nature of DL makes it difficult to interpret. The Shapley additive explanations (SHAP) method becomes an important tool for us to understand the model. SHAP-based interpretability analysis reveals that PM_10_, temperature, and relative humidity are the key drivers for PM_2.5_ in Jiaozuo City [37]. SHAP reveals that the 3-day rolling average, daily variation, and 1-day lag dominate the predictive power of PM_2.5_ in the UK [44]. SHAP analysis identified CO, SO_2_, and O_3_ as the key contributors to PM_2.5_ levels in Shanghai [45].

Although DL methods have made progress in PM_2.5_ forecasting, there are still problems, such as a lack of interpretability, improper network structure processing, incomplete information transmission of sparse structures, and dynamic dependency limitations. This study proposes an innovative hybrid CNN–BiLSTM–Transformer model that aims to comprehensively utilize the advantages of three architectures: CNN extracts local features, BiLSTM captures bidirectional long-term dependencies, and Transformer extracts long-term dependencies, as well as global features of input data. This multi-level feature extraction mechanism enables it to better understand the complex patterns of PM_2.5_ changes. To conduct interpretable artificial intelligence and extended analysis, the SHAP framework is used to quantify the contribution of each input variable, revealing the most influential features. The purpose of the research is to improve the prediction accuracy of PM_2.5_ and identify key influencing factors.

The main contributions of this study include the following: (1) a novel SHAP explainable CNN–BiLSTM–Transformer hybrid model is constructed for PM_2.5_ prediction; (2) the performance of the hybrid model and the single models is evaluated systematically; (3) the key factors affecting PM_2.5_ prediction are revealed through SHAP analysis; (4) practical predictive tools and scientific insights are provided for air quality management in Qingdao City.

## 2. Materials and Methods

### 2.1. Data

Qingdao is an important coastal city in China. It has a population of 10.4425 million people. In 2024, the GDP of Qingdao was 1671.946 billion yuan. Meteorological data are sourced from the platform (https://data.cma.cn, accessed on 17 July 2024), while air quality data are obtained from the platform (https://www.cnemc.cn, accessed on 21 August 2024). To ensure a realistic assessment of model generalization under operational forecasting conditions, a strict chronological split was adopted. These are divided into three parts: training part (from 1 January 2014 to 6 August 2019), validation part (from 7 August 2019 to 18 April 2020), and testing part (from 19 April 2020 to 31 December 2020), avoiding any information leakage across periods. This temporal partitioning reflects the practical scenario in which future PM_2_._5_ concentrations are predicted using only historical observations. In order to improve model performance, all variables are normalized utilizing the minimum–maximum scale method, which converts the data into a range of [0, 1]. This standardization helps the deep learning and hybrid models to more effectively handle input variables of different scales.

### 2.2. Artificial Neural Network (ANN)

ANN approximates complex nonlinear relationships between input features and the target through a series of interconnected neurons arranged in layers. The network includes fully connected hidden layers with activations and a learning rate, trained using mean squared error loss [46].

### 2.3. Recurrent Neural Network (RNN)

Unlike the ANN, RNN processes sequential inputs through recurrent units, enabling the model to retain information across consecutive time steps [47]. The network comprises two RNN layers, followed by a fully connected feedforward head with activation.

### 2.4. Convolutional Neural Network (CNN)

CNN applies convolutional filters across feature sequences to extract spatial correlations, followed by a fully connected feedforward head with ReLU activation and a pooling layer to produce a single output [48].

### 2.5. Bidirectional Long Short-Term Memory (BiLSTM)

BiLSTM captures both past and future temporal dependencies in daily PM_2.5_ concentrations. The BiLSTM processes sequential inputs through forward and backward LSTM layers, enabling the model to integrate information from both preceding and subsequent time steps [49].

### 2.6. Transformer

Transformer represents a major advancement in sequence modeling by replacing recurrence with a multi-head self-attention mechanism [50]. This design enables efficient representation of long-term temporal relationships, making the Transformer a powerful tool for time-series prediction tasks. Unlike recurrent architectures that process the input sequentially, the Transformer employs a multi-head self-attention mechanism that enables each time step to directly attend to all other steps within the input sequence. This property allows the model to more effectively learn long-range interactions among variables even when the sequence length is short, which fits the characteristics of daily air-pollution data. This module includes position encoding, multi head self-attention layer, and a feed-forward neural network. In standard Transformer architectures, the encoder–decoder structure is designed for autoregressive sequence generation. However, deterministic regression tasks, such as forecasting a single-step PM_2.5_, do not require sequential output generation. Accordingly, this study employs an encoder-only Transformer, which focuses on extracting stable and informative representations from the input sequence through multi-head self-attention and feed-forward transformations. Removing the decoder reduces computational complexity and avoids error accumulation associated with autoregressive decoding, resulting in a more efficient and robust modeling framework for environmental time-series prediction. The input meteorological and pollutant variables are projected into a latent representation, enhanced with positional encoding, and processed by multiple self-attention layers to capture intrinsic temporal dependencies and nonlinear interactions. The aggregated encoder output is then directly mapped to a scalar PM_2.5_ prediction through a regression head [41].

### 2.7. CNN–BiLSTM–Transformer

**Figure 1:**
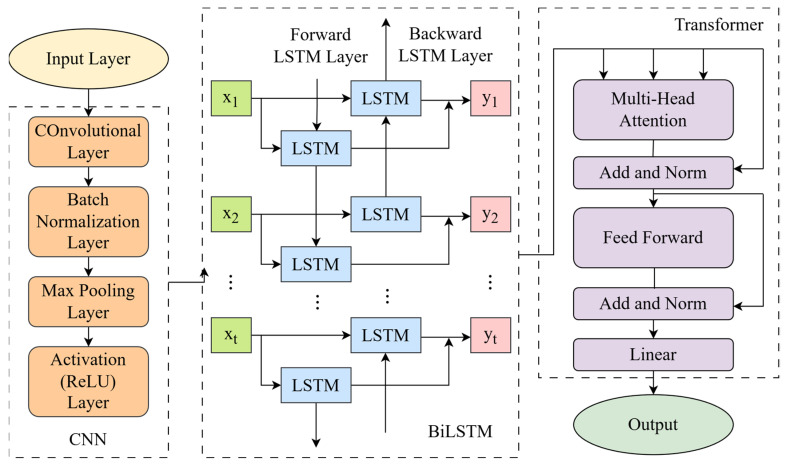
The structural diagram of CNN–BiLSTM–Transformer.

The CNN–BiLSTM–Transformer takes advantage of extracting local features and capturing both past and future context, Transformer encoder for capturing global, long-range dependencies. The hybrid CNN–BiLSTM–Transformer model can capture both local feature interactions and global temporal dependencies in daily PM_2.5_ prediction. The model first applies a stack of 1D convolutional layers to extract hierarchical local patterns across meteorological and pollutant variables. The convolutional outputs are then fed into a BiLSTM, enabling the integration of past and future temporal information, followed by a Transformer encoder that models long-range dependencies and contextual interactions. Positional encoding is applied to the Transformer inputs to retain sequence order, and the final representation is aggregated via adaptive average pooling and mapped to a single regression output through a fully connected head (Figure 1).

In this study, we adopt a modified Transformer design tailored for single-step atmospheric pollutant prediction. Specifically, we employ an encoder-only Transformer, omitting the decoder component since the task does not require autoregressive sequence generation. The input meteorological and pollutant variables are projected into a latent representation, enhanced with positional encoding, and processed by multiple self-attention layers to capture intrinsic temporal dependencies and nonlinear interactions. The aggregated encoder output is then directly mapped to a scalar PM_2.5_ prediction through a regression head. Unlike the original encoder–decoder Transformer, this regression-oriented configuration relies solely on the encoder block, which provides a computationally efficient representation learning process. The aggregated encoder output is subsequently passed through a regression head to generate the final PM_2.5_ prediction. This design allows the model to effectively exploit multi-scale temporal relationships without the overhead of a decoding module. This streamlined design improves robustness and enhances predictive performance while preserving the essential strengths of self-attention-based modeling.

We employ a one-day lag structure. The model uses daily meteorological and pollutant data from the previous day (t − 1) as input features to predict the daily average PM_2.5_ concentration for the current day (t). Therefore, all input variables are explicitly the one-day-lagged versions of the observed predictors. Within this explicit lag framework, the role of our CNN–BiLSTM–Transformer architecture is to implicitly learn the complex, nonlinear mapping from yesterday’s atmospheric state to today’s PM_2.5_ level. The model learns the interactions and relative importance among these lagged variables.

### 2.8. Shapley Additive Explanations (SHAP)

SHAP calculates the contribution of each feature to the model output, providing a fair interpretation for each data point. It not only reveals the impact of individual features on prediction, but also allows us to better understand the interactions between features.

To interpret the relative influence of meteorological and pollutant predictors on the hybrid CNN–BiLSTM–Transformer model, we use the SHAP framework. SHAP provides an additive and locally accurate decomposition of the model output:

$$f(x)=\emptyset_{0}+\sum_{i=1}^{d}\emptyset_{i\;}$$

where $\emptyset_{0}$ represents the expected model prediction and $\emptyset_{i\;}$ denotes the marginal contribution of predictor i across all possible feature combinations. This game-theoretic formulation ensures consistency and enables transparent interpretation of nonlinear and high-capacity models commonly applied in PM_2.5_ predictions. Overall importance is summarized using the mean absolute SHAP value:

$$Mean\left|\emptyset_{j}\right|=\frac{1}{m}\sum_{m}^{1}\left|\emptyset_{i,j}\right|$$

This interpretability approach offers a robust, model-agnostic assessment of how meteorological and emission-related variables shape the model’s PM_2.5_ predictions, strengthening the transparency and policy relevance of the analysis [51].

### 2.9. Evaluation Indices

**Figure 2:**
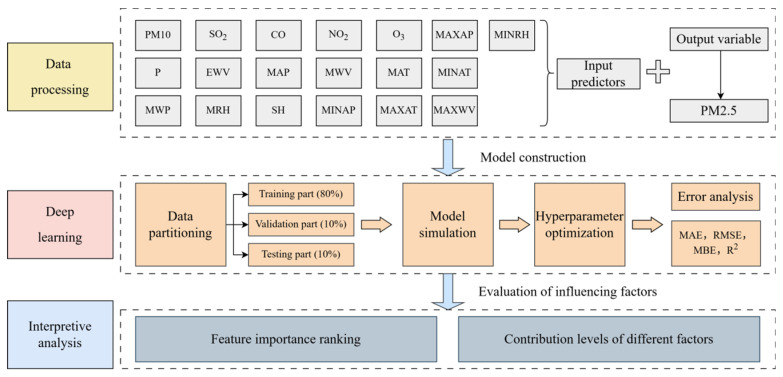
The technical roadmap.

To evaluate the accuracy of the deep learning and hybrid models, several performance indices are employed, including R, RMSE, MAPE, and MAE [52]. The calculation formulas are as follows:

$$R=\frac{\sum_{i=1}^{n}(x_{i}-\bar{x})(y_{i}-\bar{y})}{\sqrt{\sum_{i=1}^{n}(x_{i}-\bar{x}{)}^{2}\sum_{i=1}^{n}(y_{i}-\bar{y}{)}^{2}}}$$

$$MAE=\frac{1}{n}\sum_{i=1}^{n}|x_{i}-y_{i}|$$

$$RMSE=\sqrt{\frac{1}{n}\sum_{i=1}^{n}(x_{i}-y_{i}{)}^{2}}$$

$$MAPE=\frac{100}{n}\sum_{i=1}^{n}\left|\frac{x_{i}-y_{i}}{x_{i}}\right|$$

where $x_{i}$ represents the actual PM_2.5_ concentrations, $y_{i}$ represents the predicted PM_2.5_ concentrations, $\bar{x}$ and $\bar{y}$ are, respectively, the mean of the actual and forecasted values, and n is the total number of observations. The technical roadmap is shown in Figure 2.

## 3. Results

### 3.1. Correlation Between Input Variables and PM_2.5_

To establish deep learning prediction models, we analyze the relationship between the input predictors and PM_2.5_ (Table 1). The order of R for each predictor is PM_10_, CO, NO_2_, SO_2_, MINAT, MAT, MAXAT, and MWP. The correlation between PM_2.5_ and PM_10_ is the highest, followed by CO, NO_2_, and SO_2_. Among meteorological elements, PM_2.5_ has the highest correlation with MINAP, followed by MAT, MAXAT, and MWP. PM_2.5_ is positively correlated with MAP, MINAP, and MAXAP, and negatively correlated with other meteorological predictors. These results confirm the substantial impact of predictors on PM_2.5_. Therefore, 19 predictors are selected as input variables for deep learning and hybrid models.

### 3.2. Hyperparameter Optimization

The optimized hyperparameters of deep learning models mainly include learning rate, optimizer, batch size, and iteration cycle based on grid search (Table 2). The unit of hidden layers in deep learning models is 64. The number of iterations is 100, the learning rate is 0.0001, the CNN kernel size is 3 × 1, the batch size is 32, the convolutional filters are 64 and 96, the max pooling is 2 × 1, and the Adam optimizer is used for the deep learning models. The hyperparameters of the hybrid model are combinations of individual models. The hyperparameters of the CNN–BiLSTM–Transformer model are as follows: the kernel size of the convolutional layer is 3 × 1, the convolutional filters are 64 and 96, the max pooling is 2 × 1, the unit of the hidden layer in BiLSTM is 64, the learning rate is 0.0001, and the batch size is 32. The hidden layer dimension (embedding dimension) of the Transformer is 128, the number of heads with attention is 8, the number of encoder layers is 3, and the dropout rate is 0.1.

### 3.3. Performance Comparison of the Deep Learning and Hybrid Models

Table 3 shows the performance of the deep learning and hybrid models using the training, validation, and test datasets along with key statistical performance metrics, including R, MAE, MAPE, and RMSE. The hybrid CNN–BiLSTM–Transformer model for forecasting PM_2.5_ performs the best in all evaluation metrics. The hybrid CNN–BiLSTM–Transformer model is superior to single CNN, BiLSTM, and Transformer. Compared to other models, the CNN–BiLSTM–Transformer model had the lowest RMSE and MAE values.

For the training dataset, the hybrid CNN–BiLSTM–Transformer model showed high prediction accuracy with R (0.9832), indicating an almost perfect linear relationship between predicted PM_2.5_ values and actual PM_2.5_ values. The MAE is 4.6801 μg/m^3^, while the MAPE is 20.2955%, highlighting the robustness of the model. In addition, the RMSE of 6.5734 μg/m^3^ demonstrates the high accuracy of the hybrid CNN–BiLSTM–Transformer model during the training phase. However, the corresponding R values are, respectively, 0.9638 for CNN, 0.9627 for BiLSTM, and 0.9655 for Transformer. The corresponding RMSE values are, respectively, 10.8414 μg/m^3^ for CNN, 11.3422 μg/m^3^ for BiLSTM, and 10.4600 μg/m^3^ for Transformer.

For the validation dataset, the hybrid CNN–BiLSTM–Transformer model maintained relatively strong predictive performance, despite slightly higher error values compared to the training data. The R value is 0.9687, MAE is 5.6748 μg/m^3^, MAPE is 18.1750%, and RMSE is 8.1896 μg/m^3^. These values are very consistent with those observed in the validation set, providing evidence that the hybrid CNN–BiLSTM–Transformer model effectively generalizes unseen data during the validation process.

On the independent testing dataset, the hybrid CNN–BiLSTM–Transformer model maintained strong predictive performance, as expected by independent validation. The R value of the hybrid CNN–BiLSTM–Transformer model is 0.9743, while the MAE and MAPE are 4.0220 μg/m^3^ and 22.7791%, respectively, indicating a slightly lower prediction error compared to Transformer. Similarly, RMSE is observed at 5.4236 μg/m^3^, indicating a lower expansion of the error distribution compared to Transformer. The performance between the training, validation, and testing subsets is very close, indicating that the hybrid CNN–BiLSTM–Transformer model effectively generalizes, and high prediction accuracy is not the result of overfitting. However, the corresponding R values are, respectively, 0.9711 for CNN, 0.9709 for BiLSTM, and 0.9712 for Transformer. The corresponding RMSE values are, respectively, 5.9106 μg/m^3^ for CNN, 6.1696 μg/m^3^ for BiLSTM, and 5.6769 μg/m^3^ for Transformer during the predicting period.

Both CNN–BiLSTM–Transformer and Transformer models have achieved powerful forecast performance, with an R value exceeding 0.9. Compared with the Transformer, the CNN–BiLSTM–Transformer model achieves slightly higher R and shows slightly higher overall accuracy in MAPE. Its outstanding performance may be attributed to its robustness to noise and ability to capture nonlinear mappings. Although the Transformer model has slightly lower accuracy, it still has competitiveness. It effectively simulates the time structure in the data, which may be more advantageous in more complex environments. This comparison highlights the potential of data-driven time series models for PM_2.5_ predictions.

**Figure 3:**
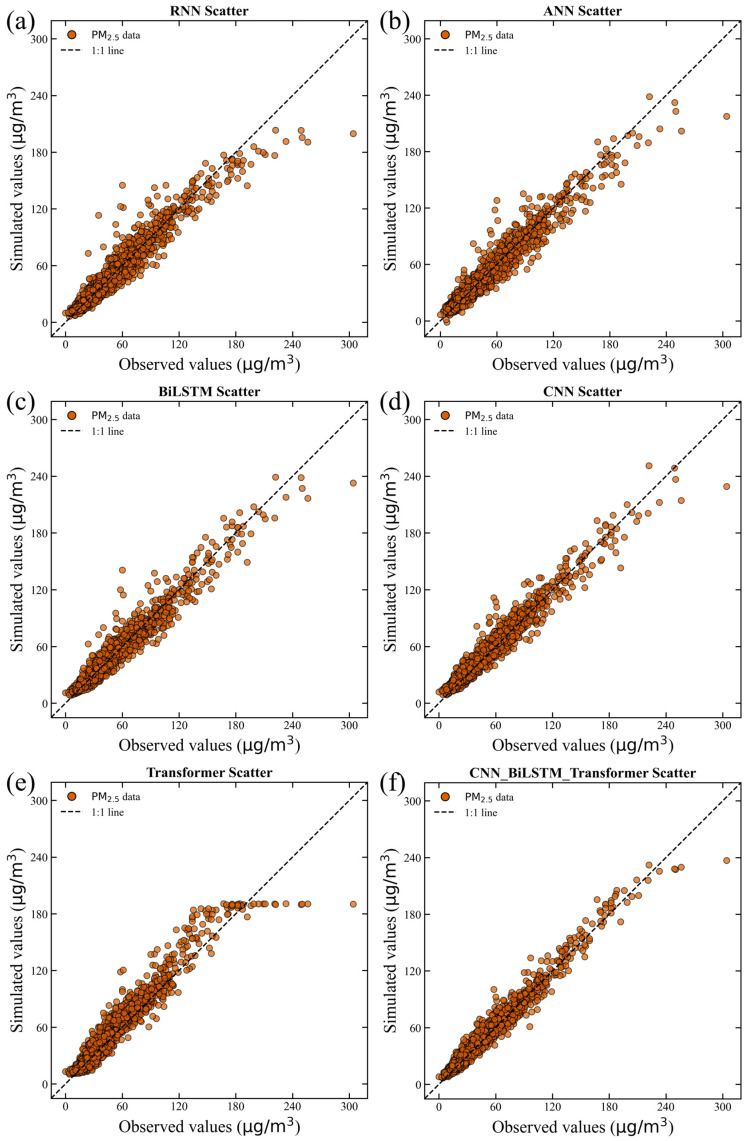
Scatter plot of the simulation results for different models during the training period. (**a**) the simulated results of RNN, (**b**) the simulated results of ANN, (**c**) the simulated results of BiLSTM, (**d**) the simulated results of CNN, (**e**) the simulated results of Transformer, (**f**) the simulated results of CNN-BiLSTM-Transformer.

**Figure 4:**
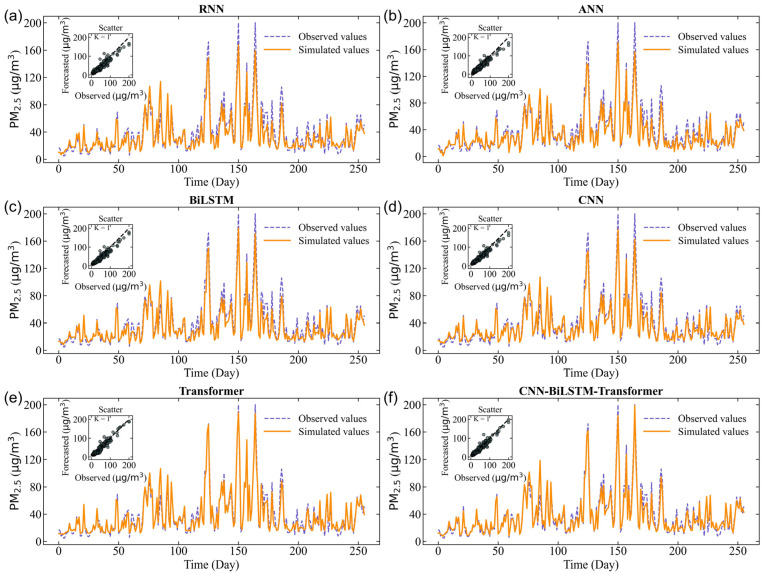
The simulation results for different models during the verification period. (**a**) the simulated results of RNN, (**b**) the simulated results of ANN, (**c**) the simulated results of BiLSTM, (**d**) the simulated results of CNN, (**e**) the simulated results of Transformer, (**f**) the simulated results of CNN-BiLSTM-Transformer.

**Figure 5:**
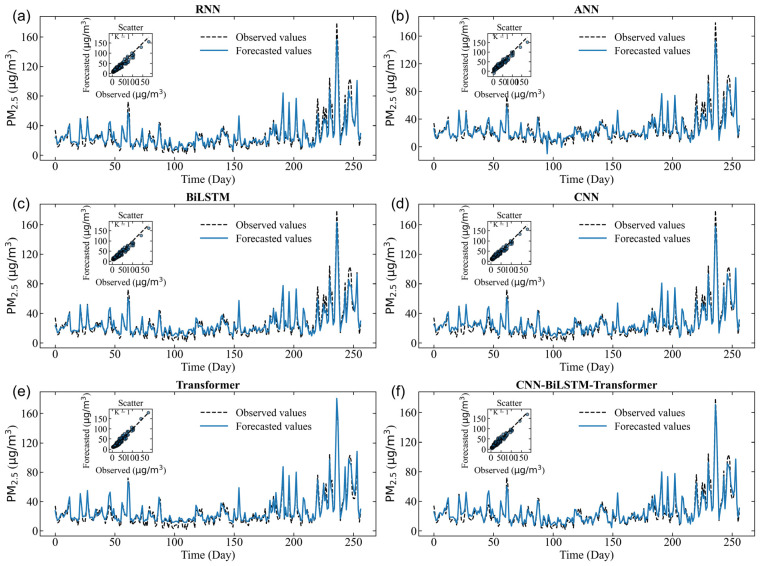
The forecasted results for different models during the testing period. (**a**) the forecasting results of RNN, (**b**) the forecasting results of ANN, (**c**) the forecasting results of BiLSTM, (**d**) the forecasting results of CNN, (**e**) the forecasting results of Transformer, (**f**) the forecasting results of CNN-BiLSTM-Transformer.

The simulated PM_2.5_ concentrations using deep learning and hybrid models in the training, validation, and test datasets are shown in Figure 3, Figure 4 and Figure 5. Although the performance metrics in Table 3 indicate only slight differences between the performance of all prediction models, Figure 3 depicts a more nuanced picture. All forecast models have successfully simulated the long-term temporal trend of PM_2.5_ concentrations. The scatter plot and line graph of the RNN model show high bias, with loosely distributed points underestimating PM_2.5_ values, indicating weak ability to capture nonlinear input–output relationships (Figure 3a, Figure 4a and Figure 5a). Similarly, ANN, BiLSTM, CNN, Transformer, and CNN–BiLSTM models also underestimated PM_2.5_ values (Figure 3b–e, Figure 4b–e and Figure 5b–e). In contrast, the scattering points of the CNN–BiLSTM–Transformer model are closest to the diagonal, and the observation points and simulation points almost coincide (Figure 3f, Figure 4f and Figure 5f). During the training, validation, and prediction stages, the CNN–BiLSTM–Transformer method outperforms other deep learning models in terms of simulation performance.

The ability of the hybrid CNN–BiLSTM–Transformer model has significantly improved in generating PM_2.5_ peaks, indicating that the hybrid CNN–BiLSTM–Transformer model helps alleviate the imbalance problem by increasing the extreme cases in the training set. This improvement indicates that the modification of the hybrid CNN–BiLSTM–Transformer model enables the model to better analyze and respond to complex patterns related to extreme PM_2.5_ pollution events. In summary, applying the hybrid CNN–BiLSTM–Transformer model helps the model make better predictions, especially for extreme PM_2.5_ pollution events, while also slightly improving the accuracy of normal events.

Specifically, the performance of the hybrid CNN–BiLSTM–Transformer is evaluated during heavy pollution in winter. Despite the significant fluctuations in PM_2.5_ during this period, the CNN–BiLSTM–Transformer is still able to capture the changing trend well, with only a slight underestimation at the peak concentration. This indicates that CNN–BiLSTM–Transformer has a certain predictive ability for extreme pollution events, but there is still room for improvement. The hybrid CNN–BiLSTM–Transformer model always provides accurate predictions. On the other hand, the RNN models seem somewhat inconsistent. Overall, the research results indicate that the hybrid CNN–BiLSTM–Transformer has the potential for practical deployment in air pollution prediction.

### 3.4. Interpretability Analysis

**Figure 6:**
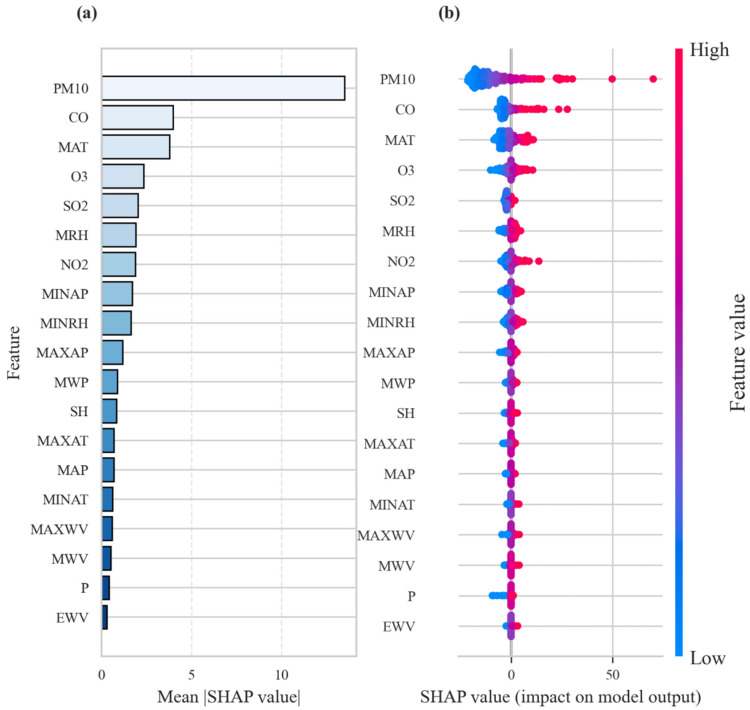
Feature importance analysis based on the hybrid CNN–BiLSTM–Transformer model. (**a**) SHAP summary plots, (**b**) SHAP mean importance plots.

SHAP is utilized for feature importance analysis of the hybrid CNN–BiLSTM–Transformer model (Figure 6). PM_10_, CO, MAT, O_3_, SO_2_, and MRH are identified as the top six factors affecting PM_2.5_ concentrations. This result highlights their crucial role as the main factors in PM_2.5_ changes. After these key predictive factors, the effects of NO_2_, MINAP, and MINRH are moderate. EWV and precipitation are the least influential features in this model. The difference in the importance ranking of other features is very small. These results indicate that specific air pollutants (PM_10_, CO, O_3_, and SO_2_) are the main drivers of PM_2.5_ concentrations, highlighting the need for targeted emission reduction measures.

### 3.5. Visualizing Feature Contributions

**Figure 7:**
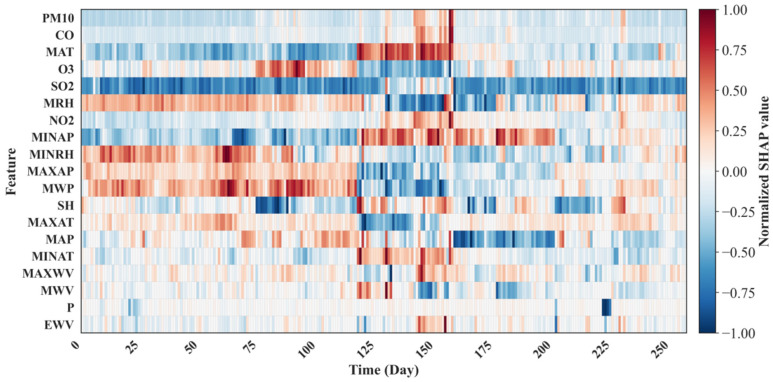
SHAP heatmap of the hybrid CNN–BiLSTM–Transformer model.

Figure 7 shows the SHAP heatmap, with the y-axis representing various influencing features and the x-axis representing PM_2.5_ data samples during the prediction period. The features are ranked in descending order of global importance, with the top feature having the greatest overall impact on all predicted results. PM_10_, CO, and MAT are at the top, indicating that they are the most stable and important driving factors affecting PM_2.5_ predictions on all days.

The red color indicates that features increase the prediction of PM_2.5_ (positive contribution, SHAP value > 0). While the blue indicates that features decrease it (SHAP value < 0). Color intensity indicates the magnitude of influence. In spring, MINRH, MRH, MAXAP, and MWP are the main positive factors of PM_2.5_ concentrations. In summer, O_3_ and MAT have a positive impact on the prediction. In autumn, PM_10_, CO, and NO_2_ positively influence the prediction. In winter, MINRH and MRH are key factors that significantly contribute positively to PM_2.5_ concentrations. These findings emphasize the intermittent impact of meteorological parameters on the PM_2.5_ forecast.

## 4. Discussion

The hybrid interpretable CNN–BiLSTM–Transformer model represents an important advancement in PM_2.5_ air pollution prediction. Compared with the single model (CNN, BiLSTM, Transformer), the hybrid CNN–BiLSTM–Transformer model can more comprehensively capture the complex characteristics of PM_2.5_ changes. CNN–BiLSTM–Transformer can also better understand the time relationships of PM_2.5_ data. This multi-level understanding enables the model to adapt to different PM_2.5_ pollution scenarios, ranging from steady changes to drastic fluctuations.

The sensitivity of the model to key Transformer hyperparameters (number of heads, encoder layers, and embedding dimension) would help us better understand the robustness of the proposed architecture. We performed controlled experiments by varying the following hyperparameters while keeping other settings and datasets constant: number of heads (4, 8, and 16), encoder layers (2, 3, and 4), and embedding dimensions (64, 128, and 256) (Table 4). The optimal combination of 128 embedding dimensions, 8 attention heads, and 3 encoder layers achieved the best overall performance, including the highest R, reflecting stronger agreement between predictions and observations; the lowest RMSE and MAE, indicating smaller prediction deviations; and a relatively low MAPE, demonstrating good control over percentage error. The model is sensitive to hyperparameter selection, with intermediate values (e.g., 8 heads, 128 embedding dimensions) providing the best trade-off between expressiveness and generalization. Larger or deeper configurations do not necessarily improve performance and may even degrade it, suggesting that a moderate-sized Transformer is adequate for the complexity of this task. The architecture is robust around the optimal configuration (dim = 128, heads = 8, layers = 3), as slight variations did not lead to significant performance drops, implying a stable optimal region. Through systematic sensitivity experiments, we have demonstrated that the proposed Transformer architecture exhibits a clear dependence on key hyperparameters for PM_2.5_ prediction. The identified optimal configuration provides an effective balance between model capacity and generalization. This analysis not only validates the design choices but also offers practical guidance for hyperparameter selection in similar temporal prediction tasks.

To specifically evaluate model performance during extreme PM_2.5_ pollution events, we identified the top 10% of observed PM_2.5_ concentrations (exceeding the 90th percentile) within the independent test set as a “High PM_2.5_ Events” subset. The CNN–BiLSTM–Transformer model’s performance on this subset was compared against its overall performance on the entire test set (Table 5). The model retains a strong correlation (R = 0.9621) even for extreme PM_2.5_ events, demonstrating its fundamental capability to capture the primary drivers and temporal patterns of severe pollution episodes. As expected for higher magnitude predictions, the absolute errors (RMSE, MAE) increase for the high-concentration subset. This is a common characteristic in regression tasks. However, the more informative metric for management, the MAPE, shows a significant improvement, decreasing from 22.7791% to 11.5314%. The substantially lower MAPE for extreme PM_2.5_ events indicates that the model’s relative prediction accuracy is actually higher during critical high-pollution periods. This is a crucial strength for practical applications, as it provides reliable proportional estimates when PM_2.5_ concentrations are most hazardous, directly supporting the issuance of accurate health alerts and management interventions. This focused assessment confirms that the proposed model is not merely optimized for general performance but is particularly robust in quantifying high-concentration PM_2.5_ events. Its superior relative accuracy (MAPE) during extremes enhances its practical utility for forecasting systems aimed at mitigating public health risks during severe pollution episodes.

Meteorological factors and pollutants affect PM_2.5_ variations. The critical driving factors are PM_10_, CO, mean atmospheric temperature, O_3_, SO_2,_ and mean relative humidity based on SHAP analysis in the study. During high temperatures, air temperature affects the diffusion of pollutants and their chemical reactivity, leading to the formation of secondary PM [53]. Rainfall removes PM from the air through wet deposition. However, the accumulation of PM_2.5_ may intensify in the absence of rainfall [54]. Through photochemical reactions, solar radiation has a crucial effect on atmospheric chemistry [55]. Longer sunshine hours increase the production of secondary pollutants, leading to the formation of PM_2.5_. Solar radiation accelerates the decomposition of precursor gases such as NO_2_ and VOC, forming O_3_. Ozone can interact with PM, leading to an increase in PM_2.5_ concentrations [55]. Relative humidity plays a dual role in the formation and persistence of PM_2.5_. Although lower humidity promotes the diffusion of pollutants and reduces the formation of particulate matter, extremely high humidity promotes the growth of aerosols, especially when combined with high levels of precursor gases such as SO_2_ and CO [54,56]. Air pressure plays a crucial role in atmospheric pollution by affecting the stability of the atmosphere [57]. High-pressure systems can enhance the diffusion ability of particulate matter, thereby reducing the concentration of particulate matter. Wind speed affects the transport and diffusion of pollutants. During low wind speeds, pollutants accumulate in the air, leading to an increase in PM_2.5_ concentration. However, as wind speed increases and affects diffusion, leading to a decrease in PM_2.5_ concentrations [56,58].

PM_2.5_ is a part of PM_10_, so changes in PM_10_ concentration usually directly affect PM_2.5_. PM_10_ and PM_2.5_ share common sources, such as coal-fired emissions and industrial processes, and coarse particulate matter can be converted into PM_2.5_ through physical and chemical processes [59]. CO can react with OH, increasing the formation of secondary pollutants of fine particulate matter [60]. SO_2_ can oxidize to form secondary particulate matter sulfates [61]. High levels of O_3_ promote the formation of SOA, leading to high levels of PM_2.5_. O_3_ is also a key oxidant in the air, affecting the formation of PM_2.5_ from precursor pollutants [54,62]. CO, SO_2_, and NO_2_ are mainly produced by the combustion of fossil fuels. Therefore, it is urgent to reduce the emissions of major pollutants (CO, SO_2_, and NO_2_). Pollution generated by transportation and factories can be reduced by implementing higher emission standards.

From a practical application perspective, the predictive model developed in this study can provide a powerful tool for air quality management in Qingdao City. The environmental protection department can use CNN–BiLSTM–Transformer to predict PM_2.5_ concentration one day in advance, providing decision support for formulating pollution control measures. In addition, accurate PM_2.5_ prediction can also be used for public health protection, especially for providing timely health advice to sensitive populations.

This study has some limitations. Firstly, only meteorological factors and historical air pollutant data were considered, and other important factors such as emission source data and regional transmission effects were not included. Secondly, there is still room for improvement in the predictive accuracy of the model in extreme pollution events.

## 5. Conclusions

This study developed a novel hybrid SHAP explainable CNN–BiLSTM–Transformer model for predicting daily PM_2.5_ concentrations in Qingdao City. With the help of SHAP values, the contribution of input features is quantified, and the “black box” of the hybrid model is interpretable. The hybrid CNN–BiLSTM–Transformer model shows superior performance in PM_2.5_ prediction tasks, significantly superior to single models (RNN, ANN, CNN, BiLSTM, Transformer), with lower error values. The model shows stable predictive ability in different seasons, especially in autumn and winter, demonstrating its adaptability to different meteorological conditions. SHAP-based interpretability analysis identified the most contributors to PM_2.5_ levels, including PM_10_, CO, MAT, O_3_, SO_2,_ and MRH, providing new insights into understanding the mechanisms of PM_2.5_ changes. The SHAP method is adopted to improve the interpretability of deep learning, providing a powerful framework for data-driven air pollution prediction. This hybrid CNN–BiLSTM–Transformer model has potential applications in actual air quality management, providing a scientific basis for pollution control and public health protection.

In the future, we can apply this hybrid interpretable model to other places to improve its practicality and applicability in different urban environments. We will integrate more data sources, such as emission inventories and satellite remote sensing data. Considering the complexity of chemical and physical processes of pollutants, we hope to achieve a nonlinear representation of data through deep learning methods and explore the complex spatiotemporal relationships between pollutant emissions. We will also explore more advanced self-attention mechanisms and dynamic feature selection methods, optimize model structures, and further improve PM_2.5_ prediction accuracy, especially in extreme pollution events.

## Figures and Tables

**Table 1:** Correlation between PM_2.5_ and influencing factors.

Influence Factor	Abbreviation	R
PM_10_	PM_10_	0.8995
SO_2_	SO_2_	0.6110
CO	CO	0.7074
NO_2_	NO_2_	0.6692
O_3_	O_3_	−0.1478
precipitation	P	−0.1423
mean atmospheric pressure	MAP	0.2893
extreme wind velocity	EWV	−0.1459
mean atmospheric temperature	MAT	−0.3860
mean wind velocity	MWV	−0.0963
mean relative humidity	MRH	−0.0597
mean water pressure	MWP	−0.3591
minimum AP	MINAP	0.2909
sunshine hours	SH	−0.1180
maximum AP	MAXAP	0.2927
minimum AT	MINAT	−0.3934
maximum WV	MAXWV	−0.0953
maximum AT	MAXAT	−0.3672
minimum RH	MINRH	−0.1139

Minimum AP represents minimum atmospheric pressure, maximum AP represents maximum atmospheric pressure, minimum AT represents minimum atmospheric temperature, maximum WV represents maximum wind velocity, maximum AT represents maximum atmospheric temperature, and minimum RH represents minimum relative humidity.

**Table 2:** The hyperparameters of the models.

Hyperparameters	ANN	RNN	BiLSTM	CNN	Transformer
Units in HL	64	64	64		64
Activation function	Logsig-purelin	Tanh-sigmoid	Tanh-sigmoid	Relu	Gelu
Learning rate	0.0001	0.0001	0.0001	0.0001	0.0001
Batch size	32	32	32	32	32
Epochs	100	100	100	100	100
Optimizer	Trainbr	Adam	Adam	Adam	Adam
Kernel size				3	
Max-pooling				2	
Convolution filters				64-96	

**Table 3:** The four evaluation indicators for the models.

Models	R			RMSE (μg/m^3^)			MAE (μg/m^3^)			MAPE (%)		
	Training	Verification	Predicting	Training	Verification	Predicting	Training	Verification	Predicting	Training	Verification	Predicting
RNN	0.9612	0.9544	0.9674	11.9428	10.8593	6.3036	8.1027	7.5617	4.6586	25.4569	21.8824	34.2459
ANN	0.9617	0.9629	0.9680	11.8627	10.2998	6.2723	8.0425	7.3455	4.6529	24.7668	21.5320	33.4613
BiLSTM	0.9627	0.9661	0.9709	11.3422	9.8537	6.1696	7.6274	7.0621	4.6414	23.6251	20.9814	32.3479
CNN	0.9638	0.9677	0.9711	10.8414	9.0371	5.9106	7.4928	6.4172	4.5856	22.8975	20.9579	31.5879
Transformer	0.9655	0.9684	0.9712	10.4600	8.1992	5.6769	7.0681	5.6803	4.4915	21.1830	19.1756	31.3887
CNN–BiLSTM–Transformer	0.9832	0.9687	0.9743	6.5734	8.1896	5.4236	4.6801	5.6748	4.0220	20.2955	18.1750	22.7791

**Table 4:** The sensitivity of the CNN–BiLSTM–Transformer model to key Transformer hyperparameters.

Embedding Dimension	Number of Heads	Encoder Layers	R	RMSE (μg/m^3^)	MAE (μg/m^3^)	MAPE (%)
64	4	2	0.9684	8.7654	6.2082	24.3989
64	4	3	0.9633	9.3897	6.4147	24.1623
64	4	4	0.9629	9.3456	6.3296	24.3151
64	8	2	0.9680	8.6745	6.1392	24.4983
64	8	3	0.9670	9.5390	6.7693	24.2755
64	8	4	0.9640	9.1835	6.6503	24.9770
64	16	2	0.9662	8.8925	6.2809	24.7377
64	16	3	0.9620	9.1232	6.2082	24.8126
64	16	4	0.9682	8.8714	6.3670	24.9837
128	4	2	0.9704	8.4533	5.7435	23.7833
128	4	3	0.9730	7.8222	5.4710	23.7285
128	4	4	0.9656	8.8593	6.1632	23.8092
128	8	2	0.9681	8.4701	6.0639	23.3543
128	8	3	0.9743	5.4236	4.0220	22.7791
128	8	4	0.9665	8.4009	5.4240	23.3543
128	16	2	0.9689	8.4177	6.0283	23.6519
128	16	3	0.9725	7.9030	5.5623	23.9408
128	16	4	0.9705	8.1568	5.6960	23.0055
256	4	2	0.9677	8.2928	5.9024	23.6324
256	4	3	0.9740	7.5075	5.2639	23.1160
256	4	4	0.9742	7.8369	5.7447	23.8093
256	8	2	0.9700	8.4563	6.1401	23.8292
256	8	3	0.9710	8.4114	5.8592	23.8874
256	8	4	0.9703	8.1029	5.7008	23.7381
256	16	2	0.9730	8.3425	5.9998	23.6920
256	16	3	0.9677	8.2103	5.4124	23.0151
256	16	4	0.9709	8.1570	5.7105	23.7410

**Table 5:** The model performance for predicting extreme PM_2.5_ events during the prediction period.

Subset	R	RMSE (μg/m^3^)	MAE (μg/m^3^)	MAPE (%)
Overall test set	0.9743	5.4236	4.0220	22.7791
High PM_2.5_ events	0.9621	9.8215	8.5923	11.5314

## Data Availability

The raw data supporting the conclusions of this article will be made available by the authors on request.
